# Hand Hygiene: do we know the WHO 5 moments?

**DOI:** 10.1093/eurpub/ckac131.335

**Published:** 2022-10-25

**Authors:** J Baca Hidalgo, M Pérez Dionisio, V Salguero Cano, J Díaz Campanón, S Gutiérrez Linares, R Padilla Matas

**Affiliations:** Medicina Preventiva, Hospital Universitario Clínico San Cecilio, Guadix, Spain

## Abstract

**Background:**

The proliferation of multi-resistant bacteria to antibiotics is one of the main global health problems of the 21st century. The World Health Organization (WHO) has listed antibiotic resistance as one of the top ten public health threats facing humanity. The hands become the main vehicle for transmission by contact of the microorganisms that cause healthcare-associated infections (HAIs). Its control provides a main point in patient safety. Hand Hygiene (HH) is a good practice consolidated in the technical guides of good practices for patient safety, being essential due to its easy use, low cost and effectiveness in preventing the cross-transmission of microorganisms. Our objective is to analyze the knowledge of healthcare professionals in reference to HH according to the WHO’s indications.

**Methods:**

Descriptive study through cross-sectional surveys based on the HH Knowledge Questionnaire for healthcare professionals translated by the Health Ministry, Social Policies and Equality of Spain and validated by the WHO, carried out voluntarily and anonymously between September 2021 and March 2022, prior and after training sessions in HH.

**Results:**

There were total of 558 surveys (questionnaires) with 57% correct answers in the questionnaires before training and 62.9% after the trainning. The highest percentage of correct answers, both before and after the trainning, correspond to the HH technique and the wrong ones are related to the 4th Moment and 3rd Moment of the WHO HH.

**Conclusions/ recommendations:**

Despite being immersed in a pandemic where adherence to the correct technique of HH has been revealed, the need for healthcare professionals to carry out continuous training regarding HH and Health-care associated infection (HAI) is unquestionable.

**Key messages:**

• Hand Hygiene is a crucial good practice to control Health-care associated infection.

• Continuous trainning improves compliance in Hand Hygiene.

